# 2D layered MSe_2_ (M = Hf, Ti and Zr) for compact lasers: nonlinear optical properties and GHz lasing

**DOI:** 10.1515/nanoph-2022-0250

**Published:** 2022-06-10

**Authors:** Genglin Li, Wenhui Du, Shuo Sun, Qingming Lu, Zhixiang Chen, Hongliang Liu, Yandong Ma, Xiaoli Sun, Yuechen Jia, Feng Chen

**Affiliations:** School of Physics, State Key Laboratory of Crystal Materials, Shandong University, Jinan 250100, China; School of Chemistry and Chemical Engineering, Shandong University, Jinan 250100, China; Tianjin Key Laboratory of Micro-scale Optical Information Science and Technology, Institute of Modern Optics, Nankai University, Tianjin 300350, China

**Keywords:** saturable absorber, transition-metal dichalcogenides, ultrafast lasers, waveguide lasers

## Abstract

Two-dimensional (2D) ternary transition-metal dichalcogenides (TMDCs) are of great research interest because their superior layer-dependent optical modulation properties. In this work, three different kinds of TMDC nanosheets, including hafnium diselenide (HfSe_2_), titanium diselenide (TiSe_2_) and zirconium diselenide (ZrSe_2_), are prepared by liquid phase exfoliation (LPE) technique. The high-quality material properties of these TMDC nanosheets are confirmed by Raman spectroscopy and X-ray diffraction analysis. Furthermore, the bandgap information of five-layer MSe_2_ has been investigated via utilizing density functional theory. The calculation results exhibit ultra-narrow bandgap structure (lower than 1.1 eV) for all these three materials, indicating that MSe_2_ is suitable for broadband photonic applications. By applying the fabricated MSe_2_ as saturable absorbers, high-performance *Q*-switched mode-locked laser operation has been realized. The laser gain media are Nd:GdVO_4_ cladding waveguides fabricated by femtosecond laser direct writing. As a result, the pulsed waveguide lasers are able to deliver approximately 6-GHz laser pulses with a signal-to-noise ratio of over 45 dB. The minimum pulse width is determined to be as short as 26 ps. The results demonstrated in this work exhibit the great potential of TMDCs and waveguide structures in applications of pulsed lasers with compact footprints.

## 1 Introduction

Integrated photonics, as a fundamental for researching monolithic integration between optical devices and optoelectronic systems, plays a crucial role in promoting the emergence of quantum communication, light manipulation, and multi-functional photonic chips [1–3]. Through combining high-efficiency and broadband-response optical modulators with integrated photonic circuits, efficient on-chip modulation and fast transmission of optical signals can be realized [4–6]. Therefore, plenty of research efforts are devoted to exploring high-performance optical modulators based on all-optical [7, 8] or electro-optic solutions [9, 10]. It is widely acknowledged that two-dimensional (2D) materials, profiting by their flexible and multi-functional properties (especially ultrafast broadband optical modulation features), are extensively employed as efficient optical modulators in micro-/nano-photonics and integrated photonic devices. These layered materials are able to enrich the functionality of photonic devices and open up new possibilities for future photonic chips [11–13].

Transition metal dichalcogenides (TMDCs), as an important category of 2D materials, possess thermodynamically stable and sandwich-shaped layered structures. Therefore, these 2D materials can be fabricated into few-layer or even single-layer structures via various techniques, for instance, chemical vapor deposition (CVD) [14], pulsed laser deposition (PLD) [15], liquid phase exfoliation (LPE) [16], etc. TMDCs have the general formula MX_2_, where M represents transition metals (elements in groups IV, V or VI) and X refers to chalcogenides (S, Se or Te). Their crystal structures have the CdI_2_-type configuration in which an inner transition metal atom is coordinated by six chalcogen atoms, forming an octahedron structure [17]. Benefitting from their flexible layer structures, the bandgaps of TMDCs are generally over 1.0 eV, which can be varied from metal (multi-later) to semiconductor (monolayer) by adjusting the layer quantity [15, 18]. Their unique bandgap structures endow TMDCs with desirable optical modulation properties, which can be used as saturable absorbers (SAs) in pulsed laser generation.

Hafnium diselenide (HfSe_2_), as a newly-manufactured TMDC member, has a bandgap of around 1.11-eV, demonstrating effective optical absorption at the near-infrared (NIR) wavelengths [19, 20]. According to the previously reported work, the modulation depth of HfSe_2_ measured by *I*-scan technique is 5.8% [21], which is around 30 times higher than that of MoS_2_ [22]. Titanium diselenide (TiSe_2_) is one of the novel metal–insulator transition materials in TMDC family. It offers ultrafast nonlinear optical response and significantly compact bandgap (only 0.15 eV) [23]. Remarkably, some researchers have theoretically calculated the band structure of monolayer TiSe_2_, demonstrating a zero-bandgap structure similar to graphene and thus suggesting its great potential in broadband optical modulation applications [24]. Zirconium diselenide (ZrSe_2_) with a CdI_2_ (1T) structure presents lower than 1-eV indirect bandgap, which means that it has the potential to be utilized as a promising broadband SA in ultrafast laser generation [25, 26]. Although several bulk or fiber laser works based on these materials as SAs have been reported, there is still lacking of through investigations on their saturable absorption properties under multi-GHz pulsed laser operation. In addition, both bulk and fiber laser arrangements lack the ability of on-chip integration. As the counterpart of MSe_2_, MS_2_ (including HfS_2_, TiS_2_ and ZrS_2_) has similar crystal structures. In comparison, MS_2_ generally possesses larger bandgaps and stronger interlayer interaction originating from the higher electronegativity of sulfur element [27–29]. These properties make MS_2_ difficult to be separated into monolayer structure and limit their applications in the infrared bands [30].

In contrast to bulk and fiber lasers, waveguide lasers with compact and robust packages are highly compatible with on-chip photonic integration. Benefitting from the compact structures of optical waveguides, waveguide lasers combine the respective merits of strong mode confinement and preferable mode overlapping [31–33], thereby offering lower lasing threshold and higher intracavity optical gain compared with other laser configurations [34–37]. As a result, through combining the flexibility of waveguide structures with the desirable optoelectronic properties of 2D materials, compact pulsed lasers delivering multi-GHz pulses can be fabricated. Furthermore, by applying external modulation methods, such as electric field, on the 2D materials, tunable SA properties and thus lasing performance can be expected [38, 39]. This can be also possible for the “waveguide + 2D material” configuration by depositing micro-/nano-electrodes on the 2D material surface. A very compact strategy uses waveguide structures and 2D materials both defined on the substrate surfaces, for which the interaction between SA and guided laser modes is supported by the evanescent field [40].

In this work, we successfully fabricate HfSe_2_, TiSe_2_ and ZrSe_2_ nanosheets on sapphire substrates via LPE and spin coating methods. Their bandgaps, corresponding to the layer quantity of *N* = 5, are systematically analyzed using density functional theory (DFT). The nonlinear saturable absorption properties of these 2D layered materials are investigated by *Z*-scan and *I*-scan analysis, respectively. Modulated by these 2D layered materials, passively *Q*-switched mode-locked (QML) waveguide laser with a minimum pulse width of 26 ps and a repetition rate of 6.31 GHz is obtained.

## 2 Preparation and characterization of TMDCs

### 2.1 Thin-film TMDC preparation

Compared with other 2D layered material preparation methods, such as CVD, LPE is relatively cost-effective and less time-consuming [41]. Here, LPE technique is used for preparation of the thin-film TMDCs. In order to fabricate large-scale few-layer SAs, in the first step, mechanical milling is employed to process bulk materials (supplied by 6Carbon Technology, China) into powder for promoting the 2D material powder dissolving in solvents. Subsequently, LPE and ultrasonic centrifugation techniques are used to further reduce the layer quantity of 2D materials. The MSe_2_ (M = Hf, Ti and Zr) powder is dissolved in ethanol and sonicated for 10 h. The duration of this process depends on the interlayer interactions within materials. In general, the lower the interlayer binding energy, the shorter sonication time required [42]. Then, the MSe_2_ mixed solution is centrifuged at a fixed 10,000-rpm rotating speed for 10 min, aiming to remove impurities and ethanol insoluble fractions. We extract the supernatant of suspension, in which dissolved MSe_2_ powders possessing few-layer even monolayer morphologies, and transfer them onto the sapphire substrate surface (10 × 10 mm^2^). The obtained precipitates are placed in a vacuum oven at 60 °C for 2 h to evaporate ethanol solvent. Finally, the sample surface is spin-coated with a layer of polymethyl methacrylate (PMMA) as the protective layer to prevent the inner few-layer MSe_2_ from being oxidized. In this way, large-scale few-layer MSe_2_ SAs are successfully fabricated.

### 2.2 Materials characterization

**Figure 1:**
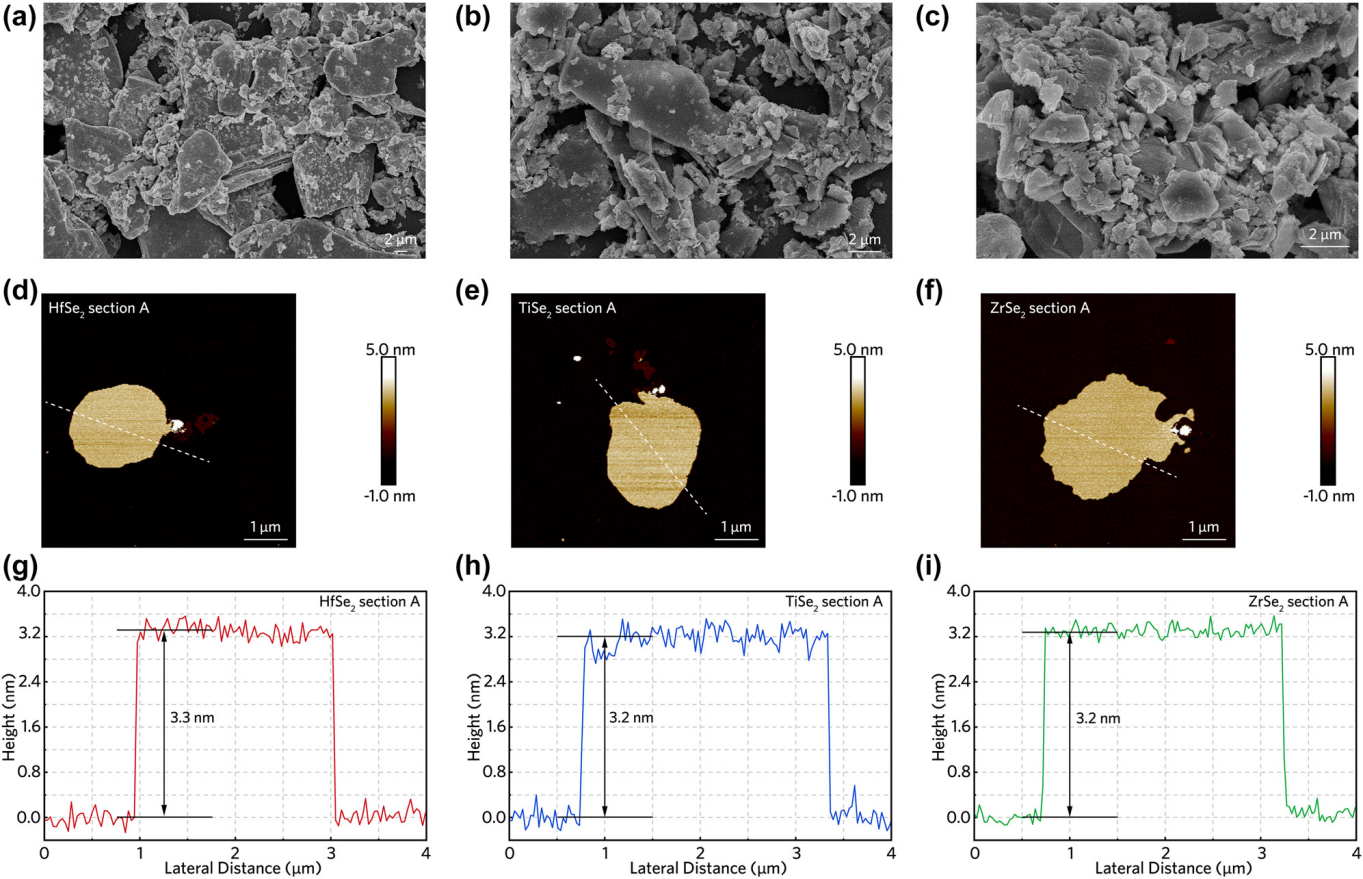
Characterization of prepared 2D MSe_2_ samples. (a)–(c) SEM images of HfSe_2_, TiSe_2_ and ZrSe_2_ powders after physical grinding. (d)–(i) AFM pictures and the height variations in white line scanning region, corresponding to HfSe_2_, TiSe_2_ and ZrSe_2_ nanosheets fabricated by LPE method.

Figure 1(a)–(c) demonstrate the morphological properties of MSe_2_ powder after physical grinding characterized by scanning electron microscopy (SEM). According to the SEM images, the geometries of the bulk materials have been significantly reduced, maintaining a horizontal size of several micrometers. The surface topography and height information of the prepared few-layer MSe_2_ nanosheets are analyzed via atom force microscopy (AFM), as illustrated in Figure 1(d)–(f). It can be observed in Figure 1(g)–(i) that the thicknesses of HfSe_2_, TiSe_2_ and ZrSe_2_ samples are 3.3, 3.2 and 3.2 nm, respectively. According to the previously reported average monolayer thicknesses of 0.62, 0.601, and 0.615 nm, respectively, for these materials [20, 43, 44], the thicknesses determined in this work are equivalent to the layer quantity of approximately five in all cases. Therefore, the processing parameters used in this work are favorable for the preparation of high-quality MSe_2_ materials with several-micrometer lateral sizes and few-layer thicknesses.

**Figure 2:**
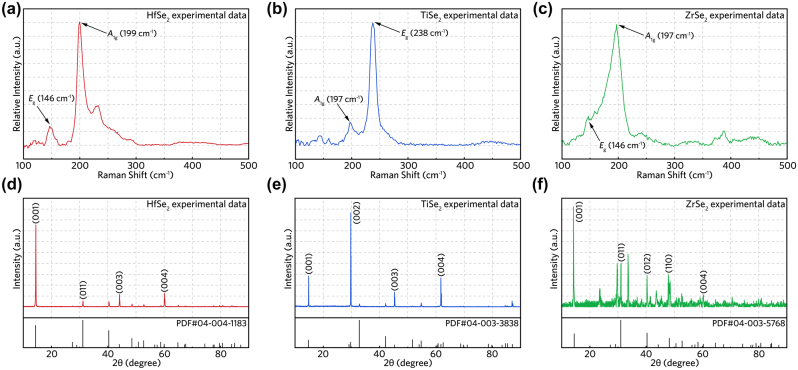
Raman spectral and XRD analysis. (a)–(c) Raman spectrums of HfSe_2_, TiSe_2_ and ZrSe_2_ sample. (d)–(e) XRD patterns of MSe_2_ samples, the colored (red, blue and green) lines are experimental data and the black line represents standard PDF card.

From the Raman spectra (see Figure 2(a)–(c)) collected from all the TMDCs prepared in this work, two typical spectral peaks, which respectively represent in-plane (*E*_g_) and out-of-plane (*A*_g_) Raman active modes, can be identified in the range of 100–300 cm^−1^ under 1064-nm laser excitation. These results are consistent with previously reported works [19, 23, 25]. The X-ray diffraction (XRD) results of MSe_2_, as demonstrated in Figure 2(d)–(f), show that the MSe_2_ powder has favorable hexagonal structure and good crystallinity, which are beneficial for the preparation of few-layer 2D materials during the subsequent LPE process. The XRD pattern of HfSe_2_ in Figure 2(d) is consist with a hexagonal structure (PDF#04-004-1183), while the strong (001) orientation is associated with a diffraction angle (2*θ*) of 14.369^°^. Compared with the other two materials, the XRD pattern of HfSe_2_ demonstrates the fewest diffraction peaks, affirming that the layer quantity of HfSe_2_ powder has been significantly reduced to few layers after grinding. The (001), (002), (003) and (004) diffraction peaks of TiSe_2_ depicted in Figure 2(e) match well with a CdI_2_ (1T) structure (PDF#04-003-3838). The typical diffraction peaks of ZrSe_2_ in Figure 2(f) are in common with 1T-ZrSe_2_ (PDF#04-003-5768), which means the lattice structure of material remain undamaged after grinding process. In comparison, more diffraction peaks in the ZrSe_2_ case could be attributed to the insufficient purity during bulk material preparation or oxidization due to the material instability and the layer stacking during characterization processes.

### 2.3 Theoretical calculation

**Figure 3:**
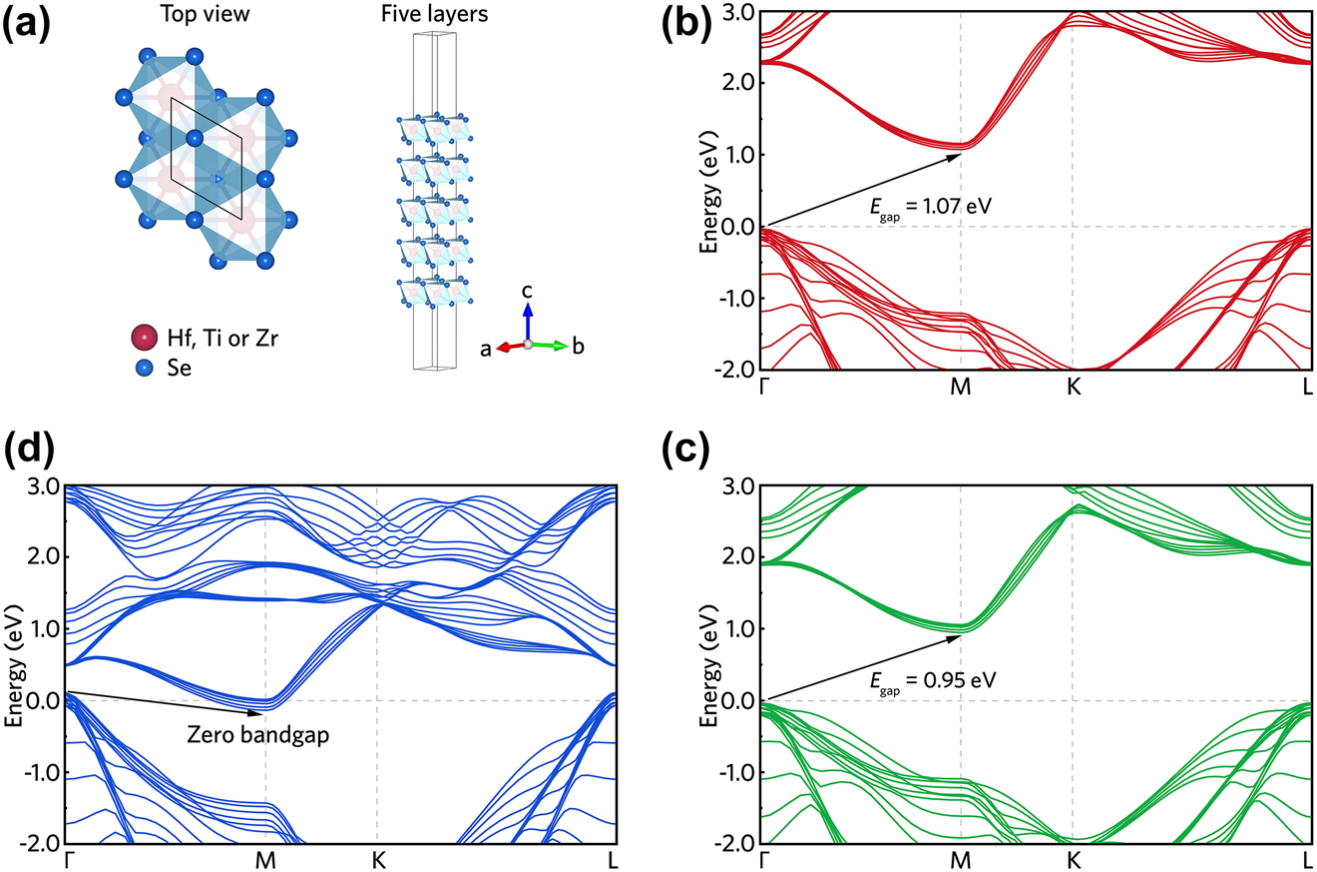
Band structure calculation. (a) Top view and crystal structure of five layers MSe_2_ materials. First-principles simulations of the five-layer MSe_2_ band structures. Calculated electronic band structures of the five-layer (b) HfSe_2_, (c) TiSe_2_ and (d) ZrSe_2_.

In order to theoretically characterize the bandgap of prepared five-layer MSe_2_ samples through LPE method (confirmed by AFM results in Section 2.2), we employ density functional theory (DFT) calculation based on the Vienna *ab initio* Simulation Package (VASP). During the calculation process, Perdew–Burke–Ernzerhof (PBE) of generalized gradient approximation function is chosen for exchange–correlation interaction. The cut-off energy of kinetic is set as 450 eV. The convergence criteria for energy and force are fixed at 10^−5^ eV and 0.01 eV/Å, respectively. A Brillouin zone is sampled by Monkhorst–Pack method with a *k*-point grid of 17 × 17 × 1. The vacuum along *z*-axis is set to 50 Å for avoiding spurious interlayer interactions. To include the strong correlation effects, the “GGA + U” method is adopted with the *U*_eff_ of 5, 3.9 and 5 eV for Hf, Ti and Zr atoms, respectively, following the previous works [29, 45, 46]. Figure 3 demonstrates the band structures of five-layer MSe_2_. We can observe that the bandgaps of HfSe_2_, TiSe_2_ and ZrSe_2_ are 1.07, 0 and 0.95 eV, corresponding to optical absorption at over 1-µm wavelengths. Thus, the calculation results confirm that all these three materials possess optical absorption properties in the NIR region. Besides the zero-bandgap material of TiSe_2_, the bandgaps of HfSe_2_ and ZrSe_2_ with different layer quantities are theoretically calculated in previously reported works [27, 29], demonstrating layer-dependent bandgaps, and thus layer-dependent optical properties.

### 2.4 Optical and nonlinear absorption properties

**Figure 4:**
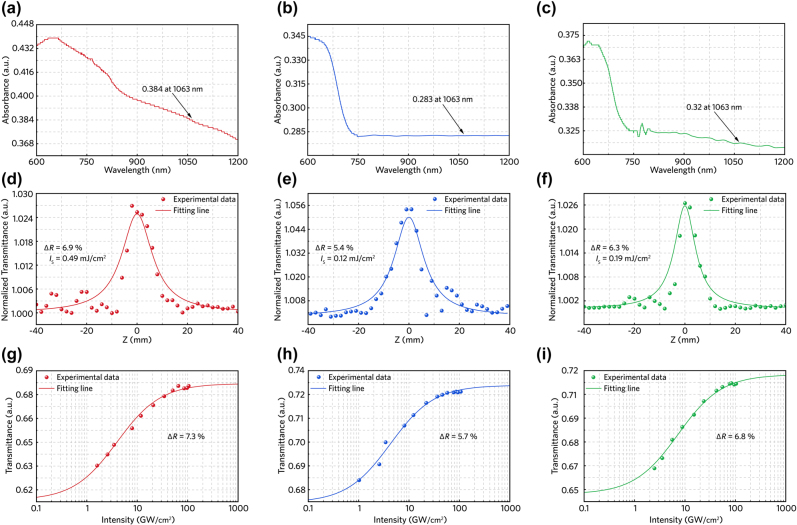
Linear and nonlinear optical properties. (a)–(c) Optical absorption spectrums of HfSe_2_, TiSe_2_ and ZrSe_2_ on sapphire substrate. (d)–(e) Open-aperture (OA) *Z*-scan results of MSe_2_ based SAs under 1035-nm laser excitation (g)–(i) The intensity dependent nonlinear transmittances of few-layer HfSe_2_, TiSe_2_ and ZrSe_2_ nanosheets at 1035 nm.

The linear optical absorption properties of the prepared samples are characterized by a Shimadzu UV-3600 spectrophotometer, as demonstrated in Figure 4(a)–(c). The optical absorbance values of the prepared MSe_2_ are all higher than 0.28 at 1.06 μm, suggesting that the potential of these 2D layered materials for nonlinear optical absorption applications. By utilizing the open-aperture *Z*-scan technique, the nonlinear optical absorption properties of the prepared MSe_2_ samples at 1.03-μm wavelength are characterized. In the home-built *Z*-scan setup, a 1035-nm mode-locked fiber laser (Femto YL-10, YSL Photonics, China) is used, which provides 400-fs pulse duration, 400-μJ maximum pulse energy and 25-kHz to 5-MHz tunable repetition rate. The laser beam with a waist radius of ∼60 μm is focused on the surface of prepared MSe_2_ samples. During the measurement, the prepared SA samples are placed at a PC-controlled motorized stage moving along the laser beam direction (*z*-axis). The incident and transmitted light power are monitored by two power meters simultaneously. As the sample moves toward the focal point (*z* = 0), the energy density irradiated on sample surface increases continuously while the normalized transmittance curve becomes sharp, which means that the optical absorption starts to saturate.

The normalized transmittance curves with approximately symmetrical shapes (centered at the focal point) are demonstrated in Figure 4(d)–(f), and the peak values at *z* = 0 position confirms that the MSe_2_-based samples possess favorable optical modulation properties. The fluctuation of the experimental data primarily originates from the inhomogeneity of material distribution on substrate surface. Nevertheless, we can obtain the modulation depth and the saturation intensity of materials through fitting the normalized experimental data (transmittance as a function of coordinate position) according to the following formula [47],

(1) $$T=\left(1-\frac{\Delta R\times I_{S}}{I_{S}+\frac{I_{0}}{1+\frac{z^{2}}{z_{0}^{2}}}}\right)/\left(1-\Delta R\right)$$

where *T* represents the normalized transmittance, Δ*R* stands for the modulation depth, *I*_0_ and *I*_s_ are the incident and saturable intensity, respectively. *Z*_0_ is the Rayleigh length of incident laser beam. As a result, the modulation depths of MSe_2_ are simulated to be 6.9% (HfSe_2_), 5.4% (TiSe_2_) and 6.3% (ZrSe_2_) and the saturable intensities are 0.49 mJ/cm^2^ (HfSe_2_), 0.12 mJ/cm^2^ (TiSe_2_) and 0.19 mJ/cm^2^ (ZrSe_2_), respectively.

Furthermore, by increasing the incident light power, the optical transmittance of the MSe_2_ materials tends to be saturated at certain values, confirming their saturable absorption properties, see Figure 4(g)–(i) (namely, *I*-scan analysis). The experimental transmittance curves are fitted by using the following formula [48],

(2) $$T=1-\frac{\Delta R}{1+\frac{I}{I_{s}}}-T_{ns}$$

where *T*, Δ*R*, *I*_s_ and *T*_ns_ are the transmittance rate, modulation depth, saturable intensity and nonsaturable loss, respectively. Under the *I*-scan model, the modulation depths are determined to be 7.3% for HfSe_2_, 5.7% for TiSe_2_ and 6.8% for ZrSe_2_. The fitting results are close to the *Z*-scan test, confirming that the MSe_2_ prepared in this work have the potential to be used as SA elements.

## 3 *Q*-switched mode-locked waveguide laser

**Figure 5:**
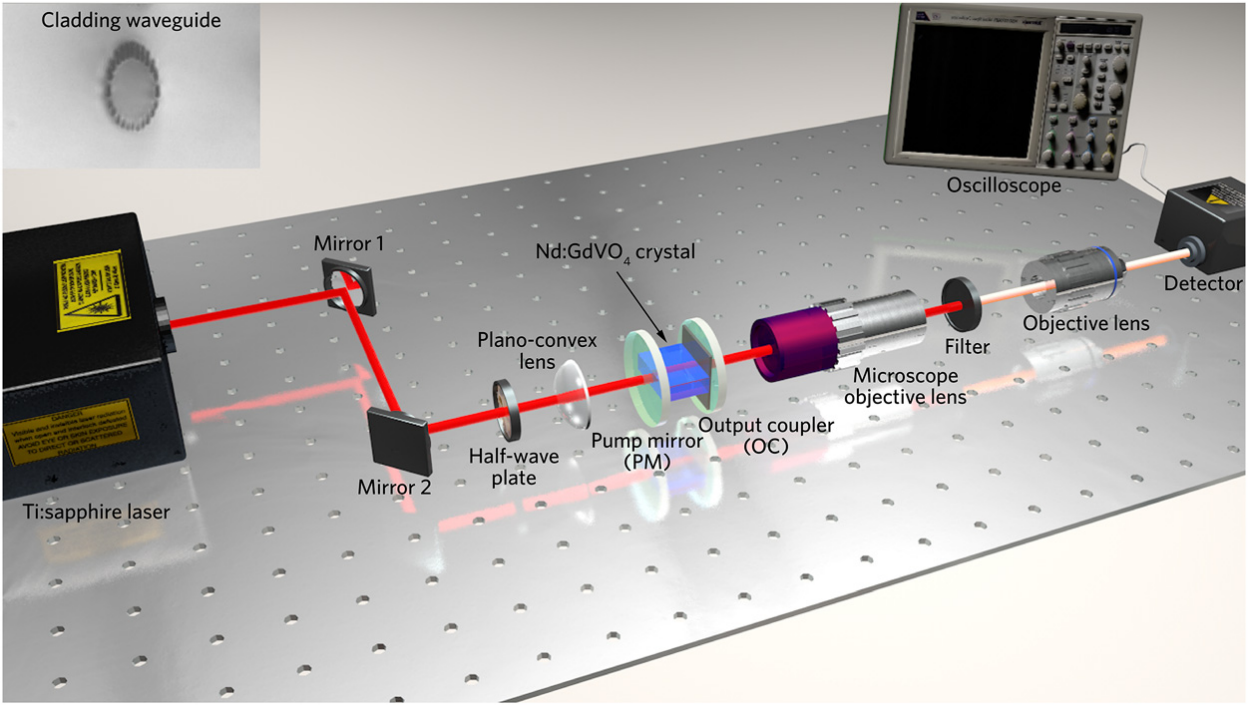
Schematic diagram of the typical end-face coupling facility for pulsed laser delivery. The inset is waveguide structure (upper-left).

In order to experimentally verify the saturable absorption properties of the prepared MSe_2_ samples, we utilized them as SAs in a waveguide laser configuration. The active gain medium used here is a Nd:GdVO_4_ cladding waveguide fabricated by FsLDW (the waveguide loss information can be found in the Supplementary material), detailed information of this technique for waveguide fabrication can be referenced elsewhere [33]. The pulsed laser generation has been investigated by a typical end-face coupling system, as illustrated in Figure 5 (the inset shows the cross-sectional image of the fabricated waveguide structure), in which a tunable continuous wave (CW) Ti:sapphire laser (Coherent MBR-110) is employed for optical pumping. The linearly-polarized 813-nm pump light is applied for the generation of 1063-nm pulsed laser. By means of combining a plano-convex lens (with a focal length of 25 mm) and a microscope objective lens (20×, N.A. = 0.4), the pump light and generated pulsed laser can be coupled into/out of the cladding waveguide. For the *Q*-switched mode-locked waveguide laser configuration, the Fabry–Perot cavity is mainly composed by three elements, namely, a pump mirror (PM), the laser crystal sample (attached with SAs), and an output coupler (OC). The PM provides a high transmittance of >99.8% at 808 nm and a high reflectivity of >99.99% at 1064 nm. The inner facet of OC coated by a part-reflection layer maintains around 40% at 1064 nm, whereas the outer surface of it provides anti-reflection (<0.25%) at 1064 nm. A long-pass filter (Thorlabs, FEL 900) is applied to eliminate the residual pump light. The filtered laser is collected by another objective lens (25|× N.A. = 0.4), aiming to couple the pulsed laser into a single-mode fiber connected directly to the High-Speed Fiber-Optic Photodetector (New focus, 1414 model). Each component in the waveguide laser setup is placed on three five-dimensional translation optical stages separately, ensuring the relative position of each part can be flexibly adjusted to achieve the best coupling conditions. Ultimately, the captured *Q*-switched mode-locked laser is observed on a digital oscilloscope (Tektronix, MSO 72504DX).

### 3.1 *Q*-switched mode-locked laser operated by HfSe_2_

**Figure 6:**
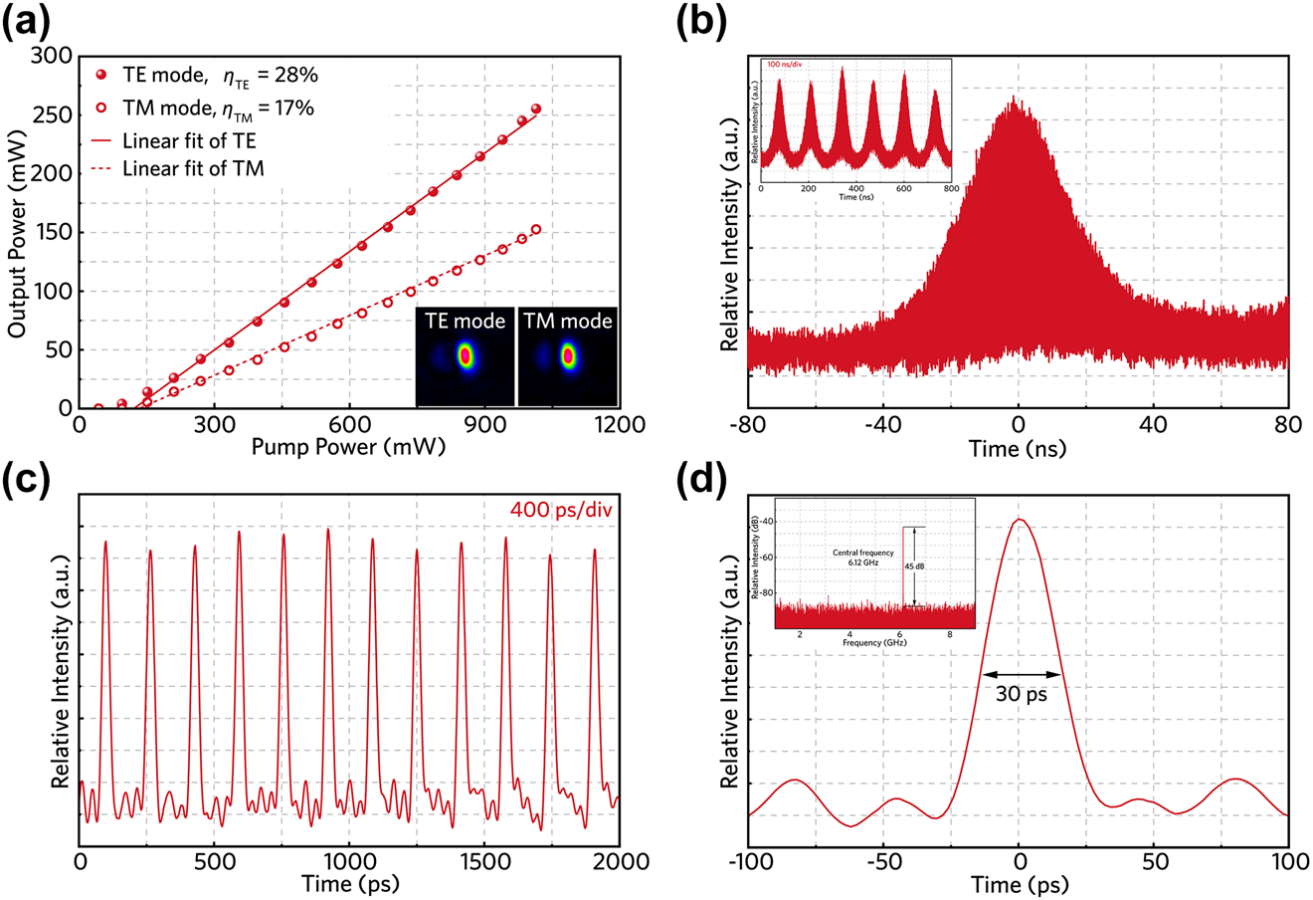
*Q*-switched mode-locked laser generated by inserting HfSe_2_ as SA. (a) The dependence between the pump power and the output pulsed laser power. The inset depicts the pulsed laser modes with TE and TM polarizations. (b) A single *Q*-switched envelope and the inset illustrates pulse trains. (c) Mode-locked pulse sequence with picosecond timescale. (d) Single mode-locked trace of output pulsed laser. The upper-left shows the radio frequency spectrum of output pulsed laser.

To further investigate the saturable absorption response of HfSe_2_, we insert the prepared HfSe_2_ sample (approximately five layers made by LPE) between the OC and the output facet of the cladding waveguide. The *Q*-switched mode-locked laser performance based on HfSe_2_ SA has been demonstrated in Figure 6(a)–(d). The measured mode profiles of pulsed laser (see Figure 6(a) insets) confirm single-mode transmission, which is beneficial to reduce the intracavity losses. As depicted in Figure 6(a), the maximum power of pulsed laser is measured to be 255.5 mW (152.7 mW) under optical pumping with TE (TM) polarization. The *Q*-switched mode-locked laser can be achieved when the pump power is increased to 90.5 mW (94.9 mW). Figure 6(b) demonstrates the typical *Q*-switched envelope composed of mode-locked sequences delivering a pulse duration of 160 ns. The inset of Figure 6(b) displays the *Q*-switched trains on nanosecond (100 ns/div). Figure 6(c) illustrates the mode-locked trains on picosecond (400 ps/div) generated by utilizing HfSe_2_ as SA, while the measured full width half maximum (FWHM) of an individual pulse is determined to be 30 ps (see Figure 6(d)). The radio frequency (RF) spectrum in the upper-left of Figure 6(d) depicts the fundamental repetition of 6.12 GHz and the signal-to-noise ratio (SNR) of 45 dB, confirming the stability of the waveguide laser operation. It is noteworthy that our experimental results shown in Figure 6(a)–(d) have relatively narrow pulse width (∼30 ps) and high repetition frequency (over 6 GHz) simultaneously, suggesting the excellent nonlinear optical properties of HfSe_2_ SA for applications in ultrafast optical modulation.

### 3.2 *Q*-switched mode-locked laser operated by TiSe_2_

**Figure 7:**
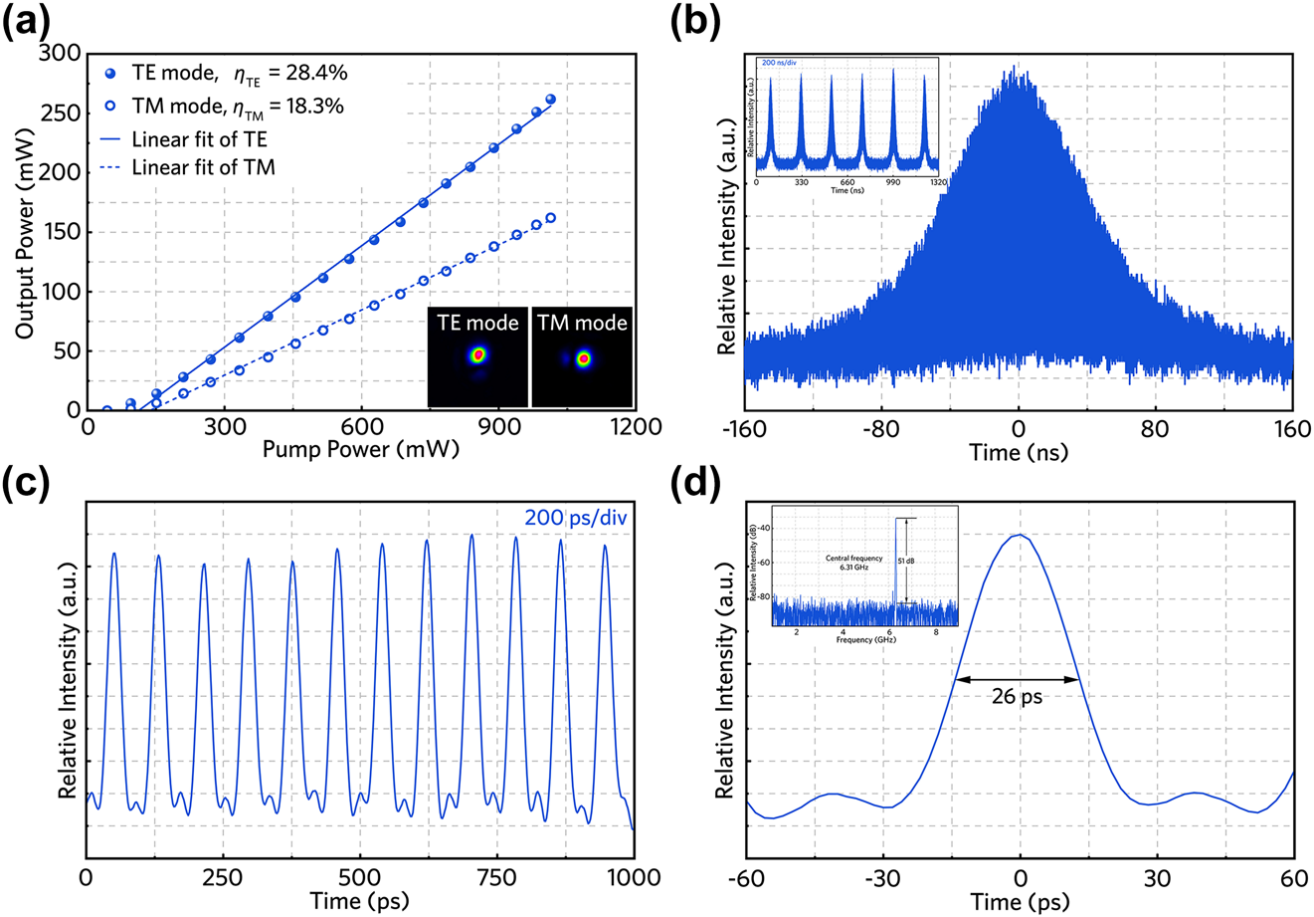
*Q*-switched mode-locked laser modulated by TiSe_2_. (a) Pulsed laser power as a function of pump power (the inset demonstrating the profiles of TE and TM modes). (b) A single *Q*-switched envelope and the inset illustrates pulse trains. (c) Mode-locked pulse sequence with picosecond timescale. (d) Single mode-locked trace of output pulsed laser and radio frequency spectrum of output pulsed laser.

Through replacing the HfSe_2_ SA by the TiSe_2_ SA, subsequently, pulsed waveguide lasers with similar performance can be achieved (see Figure 7(a)). The maximum output power is determined to be 261.9 mW (162.1 mW) and the slope efficiency is calculated to be 28.4% (18.3%) for TE (TM) mode, respectively. When the pump power exceeds over 90 mW, a stable *Q*-switched mode-locked laser can be observed. For nanosecond timescale *Q*-switched envelope demonstrated in Figure 7(b), the single pulse energy is determined to be 47.6 nJ, corresponding to a peak power of 0.48 W. Figure 7(c) and (d) illustrate the mode-locked pulse trains and the single pulse profile on picosecond timescale (200 ps/div), in which the minimum pulse width as short as 26 ps has achieved, confirming the ability of TiSe_2_ as a promising SA for ultrashort pulse laser generation. The inset of Figure 7(f) records the RF spectra of TiSe_2_ based pulsed laser, evidently, a sharp peak is identified at 6.31 GHz, corresponding to a SNR of 51 dB. During the experiment, there is no obvious optical damage to the TiSe_2_-SA.

### 3.3 *Q*-switched mode-locked laser operated by ZrSe_2_

**Figure 8:**
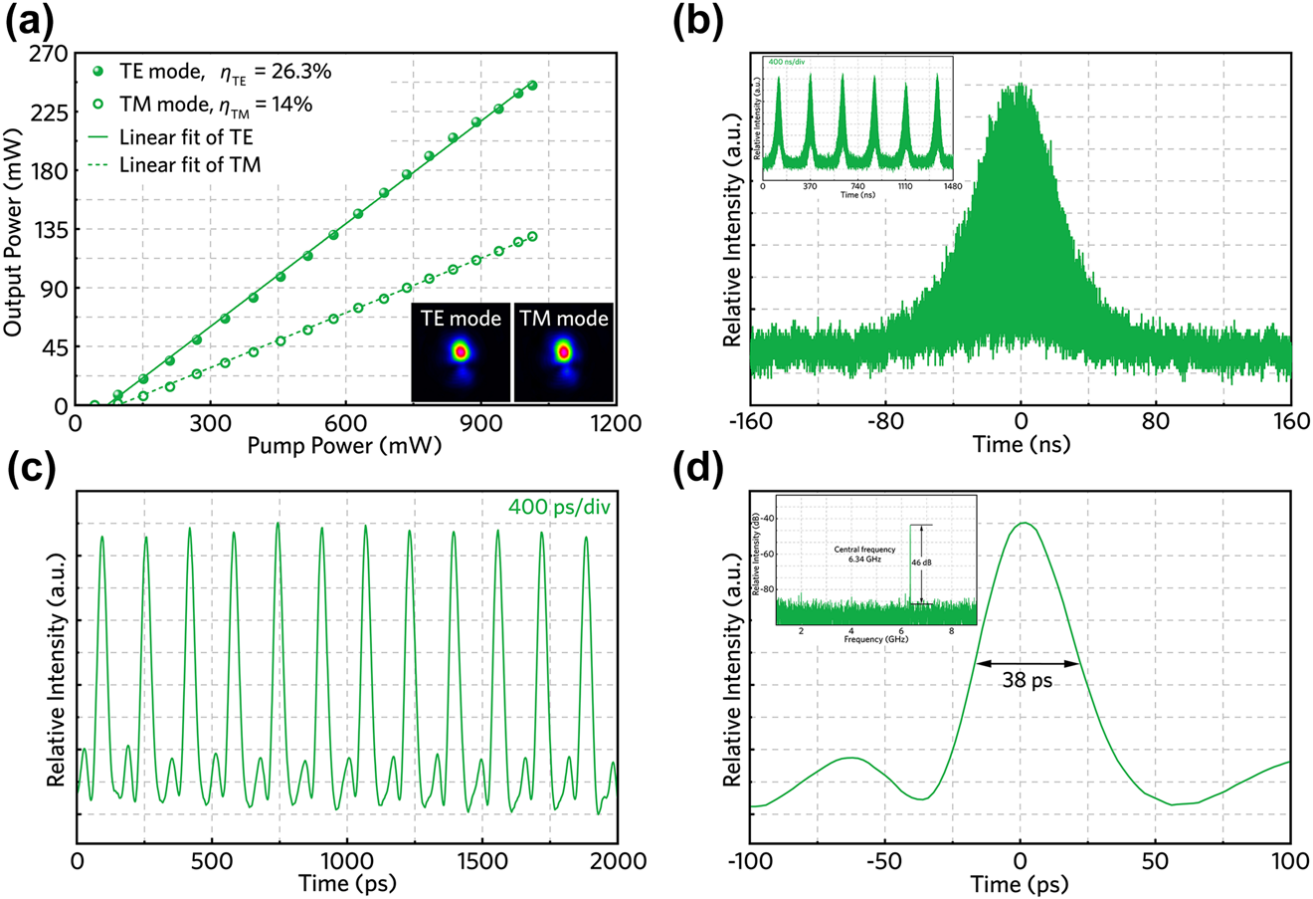
ZrSe_2_-SA based *Q*-switched mode-locked laser. (a) Linear relationship between pump power and output pulsed laser power, and the pulsed laser modes under TE and TM polarizations (down-right). (b) The measured single *Q*-switched envelope and the inset depicts the pulse trains. (c) Mode-locked pulse sequence with picosecond timescale. (d) Characterized single mode-locked trains of output pulsed laser and recorded radio frequency of the output pulsed laser.

At last, *Q*-switched mode-locked laser operation has also been investigated through inserting ZrSe_2_-SA into the waveguide cavity. As shown in Figure 8(a), single-mode laser operation has achieved in the waveguide structure when the pump power increases over 90.6 mW (95.5 mW) threshold. The maximum output power of 245.1 mW (129.4 mW) is obtained, corresponding to the pump power of 1.013 W. In contrast to the abovementioned HfSe_2_ and TiSe_2_ based lasers, the slightly inferior performance can be attributed to the partial oxidation of ZrSe_2_ sample, which has been discussed in Section 2. Figure 8(b) demonstrates a single *Q*-switched envelope composed by a serious of mode-locked pulses. The mode-locked pulse trains on the timescale of 400 ps/div are depicted in Figure 8(c). A minimum pulse duration of 38 ps is illustrated in Figure 8(d). An obvious peak located at 6.34 GHz with a SNR reaching about 46 dB is recorded in RF spectrum, as depicted in Figure 8(d).

## 4 Discussion

Up to now, several works related to HfSe_2_ SA in different laser configurations have been reported. For example, one work realized 18.1-MHz repetition rate continuous-wave mode-locked (CWML) operation in the 1.5-μm spectral region [21] and another demonstrated a *Q*-switched laser with 2.4-μs pulse width emission at 1060 nm [49]. In contrast, our experimental results shown in Figure 6(d) have a relatively narrow pulse width (∼30 ps) and a high repetition frequency (over 6 GHz) simultaneously. In addition, combining a 26-ps pulse duration with gigahertz repetition rate, the performance of *Q*-switched mode-locked waveguide laser is superior to the previously reported value of *Q*-switched Nd:GdVO_4_ laser obtained using SA based on TiSe_2_ [43]. Consequently, the approximately 6-GHz repetition rate and high-SNR (over 45 dB) waveguide laser performance in this work are comparable to those of the solid-state or fiber lasers using SAs based on TMDCs [50, 51], which means that our waveguide laser system has the potential to achieve CWML operation by further implementing efficient dispersion management [34]. Furthermore, we have also investigated the stability of the laser performance under stable optical pumping for over 1-hour time scale. As a result, only 10–15% of fluctuations on the pulse intensities can be identified for all cases, suggesting the good stability of the waveguide lasers demonstrated in this work.

The *Q*-switched mode-locked laser performance based on different TMDCs as saturable absorbers are summarized in Table 1. The fascinating pulsed laser operation based on waveguide structure can be ascribed to the excellent nonlinear optical responses of MSe_2_ (M = Hf, Ti and Zr) samples. In contrast to CVD or other material growing methods, LPE combined with spin-coating method enables the mass production of few-layer nanomaterials in a short period of time [52]. Whereas, LPE technique is hard to control the large scale (over millimeters) and consistency of prepared samples, which limits its applications in high-quality material fabrication. In addition, the compact waveguide structures offer reduced lasing thresholds and improved efficiencies. But they are suffering from absence of effective dispersion management as a result of the shortened waveguide cavities, which makes them difficult to support CWML operation.

**Table 1:** Comparisons of pulsed laser performance modulated by TMDCs.

Low-dimensional materials	Parameters					
	Prepared technic	Gain media	Laser type	Pulse width	Repetition rate	Refs.
TiSe_2_	LPE	Nd:YVO_4_	*Q*-switched	483 ns	152 kHz	[43]
MoS_2_	PLD	Nd:GdVO_4_	*Q*-switched	970 ns	732 kHz	[15]
PtSe_2_	Magnetron sputtering	Nd:LuVO_4_	CWML	15.8 ps	61.3 MHz	[60]
WS_2_	CVD	Nd:YVO_4_	*Q*-switched	39 ns	3.5 MHz	[61]
ReSe_2_	CVD	Nd:YVO_4_	CWML	29 ps	6.5 GHz	[62]
HfSe_2_	LPE	Nd:GdVO_4_	QML	30 ps	6.12 GHz	This work
TiSe_2_	LPE	Nd:GdVO_4_	QML	26 ps	6.31 GHz	This work
ZrSe_2_	LPE	Nd:GdVO_4_	QML	38 ps	6.34 GHz	This work

The “waveguide + 2D material” configuration combines flexible waveguide structures and high-performance 2D materials, offering a multi-functional platform for bridging micro-/nano-photonics and bulky systems [53]. On the one hand, waveguides with different geometries can provide high-quality optical coupling in a flexible manner. For example, the double-cladding waveguide structures with a “fiber-like” geometry show high compatibility with commercially available fibers (correlated to the fiber-waveguide coupling) [54], and the tapered waveguide structures can realize effective beam shaping (corresponding to the spatial coupling). And these are important technical points for on-chip transmission of optical signals [55]. On the other, 2D materials with ultrafast nonlinear optical response and high carrier concentration can effectively modulate the guided light [56, 57], largely enriching and improving the existing on-chip photonic functionalities [58, 59]. In future works, efforts will be made on exploring dispersion control elements (for instance, Gires-Tournois interferometer) and optimized micro-/nano-machining parameters with a view to achieving on-chip integrated CWML operation and other optoelectronic applications in the “waveguide + 2D material” configuration.

The fundamental repetition frequency for a waveguide laser based on Fabry–Perot cavity can be calculated by the following equation:

(3) $$f_{\text{repetition}}=\frac{c}{2nl}$$

where *c* is the speed of light in vacuum, *n* is the refractive index of the waveguide structure, *l* is the total length of Fabry–Perot cavity. The refractive index of 1% Nd^3+^ doped *c*-cut GdVO_4_ is approximately 2.192 at 1.06 μm [63]. Considering the total cavity length of approximately 11 mm, the fundamental repetition frequency is calculated to be ∼6.22 GHz, which is in good agreement with our experimental results. The slightly discrepancy between theoretical value and experimental results may be attributed to the unevenness of SA surface and the air gaps of intracavity.

## 5 Conclusions

In summary, we have experimentally demonstrated LPE-prepared MSe_2_ (M = Hf, Ti and Zr) as SAs for picosecond *Q*-switched mode-locked laser generation in the NIR band. In order to depict the NIR band absorption capability of prepared five-layer MSe_2_ sample, the first-principle calculation is implemented to analyze the bandgap information of MSe_2_ reported in the present work. As a result, multi-GHz repetition rate and over 45-dB SNR pulsed lasers have been obtained. The nonlinear optical responses of prepared MSe_2_ samples are investigated by open-aperture *Z*-scan and *I*-scan techniques, respectively. Other physical properties of MSe_2_ are also characterized systematically. Combined with the MSe_2_ desirable optical modulation property, 6.31-GHz *Q*-switched mode-locked laser with the minimum 26-ps pulse width has realized. The results reported in this work exhibit the excellent nonlinear optical properties of HfSe_2_, TiSe_2_ and ZrSe_2_ nanomaterials, which are very promising for construction of integrated ultrafast photonic devices.

## Data availability

Data underlying the results presented in this paper are not publicly available at this time but may be obtained from the authors upon reasonable request.

## Supplementary Material

Supplementary Material Details
