# Supplementary material for: 2D layered MSe2 (M = Hf, Ti and Zr) for compact lasers: nonlinear optical properties and GHz lasing
Source: Nanophotonics. 2022 Jun 10;11(14):3383–94. doi: 10.1515/nanoph-2022-0250 (PMC11501608; doi:10.1515/nanoph-2022-0250)
Supplement: Supplementary file 1 — Supplementary Material Details [file j_nanoph-2022-0250_suppl.docx]

Supporting Information

1. **Characterization of transmission losses in Nd:GdVO_4_ cladding waveguide**

Under the condition of single-mode operation, the transmission losses of the cladding waveguide structure can be simulated after concerning the reflectance *R* and coupling efficiency *η*. The reflectance is determined by the equation:

$R=\left(\frac{n-1}{n+1}\right)^{2}$ (S1)

where *n* is the refractive index for TE or TM polarization. The coupling efficiency is determined by the equation:

$\eta\text{=}\frac{4\omega_{1}^{2}\omega_{2}^{2}}{(\omega_{1}^{2}+\omega_{2}^{2})^{2}}$ (S2)

where *ω*_1_ and *ω*_2_ denote the diameter of focused incident 1064-nm CW laser beam and guiding mode, respectively. As a result, the transmission losses (*α*_prop._) can be calculated by the following equation:

$\alpha_{\text{prop.}}=-\frac{L}{10}\cdot\lg\left[\frac{P_{\text{out}}}{P_{\text{in}}\cdot(1-R)^{2}\cdot\eta}\right]$ (S3)

where *L* is the total length of laser cavity, *P*_in_ and *P*_out_ represent the incident and output 1064-nm CW laser power. The specific measurement and calculation results are summarized in Table S1.

**Table S1:** Summarization of the guiding properties under TE and TM polarization.

| Polarization | Refractive index (*n*) | Coupling  efficiency (%) | Incident power (mW) | Output power (mW) | Transmission losses (dB/mm) |
| --- | --- | --- | --- | --- | --- |
| TE | 2.192 | 54.1 | 186.75 | 60.84 | 0.09 |
| TM | 1.972 | 49.1 | 186.75 | 40.4 | 0.26 |
